# Design and Optimization of Intelligent High-Altitude Operation Safety System Based on Sensor Fusion

**DOI:** 10.3390/s25154626

**Published:** 2025-07-25

**Authors:** Bohan Liu, Tao Gong, Tianhua Lei, Yuxin Zhu, Yijun Huang, Kai Tang, Qingsong Zhou

**Affiliations:** 1 School of Physics, Electronics and Intelligent Manufacturing, Huaihua University, Huaihua 418000, China; 19894252045@163.com (T.G.); 18873376107@163.com (T.L.); 18059317067@163.com (Y.Z.); 13174201683@163.com (Y.H.); v2746359927@163.com (K.T.); zqsedu4216@163.com (Q.Z.); 2 Key Laboratory of Intelligent Control Technology for Wuling-Mountain Ecological Agriculture in Hunan Province, Huaihua 418000, China

**Keywords:** high-altitude operations, intelligent safety protection barrier, ultra-wideband (UWB) ranging, thin-film piezoresistive stress sensors, Beidou positioning, intelligent voice alarm, intelligent safety lock

## Abstract

In the field of high-altitude operations, the frequent occurrence of fall accidents is usually closely related to safety measures such as the incorrect use of safety locks and the wrong installation of safety belts. At present, the manual inspection method cannot achieve real-time monitoring of the safety status of the operators and is prone to serious consequences due to human negligence. This paper designs a new type of high-altitude operation safety device based on the STM32F103 microcontroller. This device integrates ultra-wideband (UWB) ranging technology, thin-film piezoresistive stress sensors, Beidou positioning, intelligent voice alarm, and intelligent safety lock. By fusing five modes, it realizes the functions of safety status detection and precise positioning. It can provide precise geographical coordinate positioning and vertical ground distance for the workers, ensuring the safety and standardization of the operation process. This safety device adopts multi-modal fusion high-altitude operation safety monitoring technology. The UWB module adopts a bidirectional ranging algorithm to achieve centimeter-level ranging accuracy. It can accurately determine dangerous heights of 2 m or more even in non-line-of-sight environments. The vertical ranging upper limit can reach 50 m, which can meet the maintenance height requirements of most transmission and distribution line towers. It uses a silicon carbide MEMS piezoresistive sensor innovatively, which is sensitive to stress detection and resistant to high temperatures and radiation. It builds a Beidou and Bluetooth cooperative positioning system, which can achieve centimeter-level positioning accuracy and an identification accuracy rate of over 99%. It can maintain meter-level positioning accuracy of geographical coordinates in complex environments. The development of this safety device can build a comprehensive and intelligent safety protection barrier for workers engaged in high-altitude operations.

## 1. Introduction

The power system constitutes the cornerstone of a country’s economic stability. Ensuring its safe and stable operation poses new challenges and requirements for the reliability of power equipment. The operation of the national power grid is generally stable, and the safety and reliability of power equipment are required [1].

With the continuous increase in the number of high-altitude power workers, safety belts play a crucial role in high-altitude operations [2]. Over the past five years, a total of 82 high-altitude fall accidents have occurred in power enterprises, representing 30.26% of all accidents. The primary cause can be attributed to employees’ non-compliance with operating procedures. Specifically, failure to wear or improperly use safety belts has been identified as the leading cause of these incidents [3]. Reference requires that the current management department, in accordance with relevant systems, stipulate that personnel engaged in high-altitude operations must wear safety belts during the construction process. However, at present, the supervision of safety belt wearing on construction sites mainly adopts the method of manual monitoring. This approach is difficult to ensure supervision efficiency in the face of the large personnel flow, dense population, and large supervision area on the construction site, and cannot guarantee the efficient implementation of safety belt supervision for high-altitude workers [4].

Experts at home and abroad have conducted in-depth research on safety devices for high-altitude operations. Li Hang, a domestic company, has designed an intelligent alarm and remote monitoring device for safety belts in high-altitude operations [5]. This method is costly, and if the 4G signal at the operation site is weak, data transmission may be delayed or interrupted, affecting the real-time performance of remote monitoring. Zhao Yinglang proposed a device with a real-time and close-range auxiliary monitoring function of “one step, one button” [6]. This device can detect in real time whether the workers at heights are wearing safety belts and issue an alarm. However, this device does not integrate a positioning module. In case of an accident, rescue personnel have difficulty obtaining accurate location information of the workers in time, which affects rescue efficiency. Yang Kuijun proposed an online intelligent safety belt detection and supervision device for high-altitude operations [7]. The overall detection speed of this method is not very ideal, and thus it cannot achieve good real-time performance. Moreover, due to factors such as weather and light, it sometimes has a certain impact on photography, and it requires monitoring by background personnel. Therefore, the entire detection will not achieve good real-time performance. Abroad, Yan Wendou proposed a YOLO-dependent Fusion Attention Network (DFAN) detection algorithm improved based on the lightweight network YOLOv4-tiny to detect whether workers in high-altitude operations are wearing safety belts [8]. This algorithm has had serious false detection and missed detection phenomena, and the safety guarantee has been significantly reduced. FANG W et al. proposed a method based on CNN to detect whether workers in high-altitude operations wear safety belts [9]. Although this method has achieved good results in terms of accuracy, due to the use of two neural networks, its real-time performance is questionable. Therefore, this paper studies a new type of safety device, which takes UWB [10] as the core component for secondary development, sets the dangerous height for high-altitude operations, and combines with induction to confirm whether it is worn correctly, making the measurement of the vertical distance from the ground simpler and more accurate. The improvement of the safety buckle makes the safety suit comfortable and safe. The efficient integration of sensors is achieved through a microcontroller. It makes the volume small and the power consumption low, achieving a transformation from passive protection to active early warning in the safety management mode, effectively solving the safety hazards caused by improper wearing of safety belts in high-altitude operations.

## 2. Key Modules and Principles of Secure Operating Systems

The safety work suit designed in this paper is composed of multiple key modules, including the UWB ranging module, the Beidou positioning module [11], the stress detection module, the intelligent safety lock, and the voice alarm module. These modules achieve the safety guarantee of the high-altitude operating system by monitoring the safety status of the operators in real time. The control core of the device adopts the STM32F103 single-chip microcomputer (STMicroelectronics Co., Ltd., Geneva, Switzerland), which is responsible for the data processing and coordination of the sensors.

**Figure 1:**
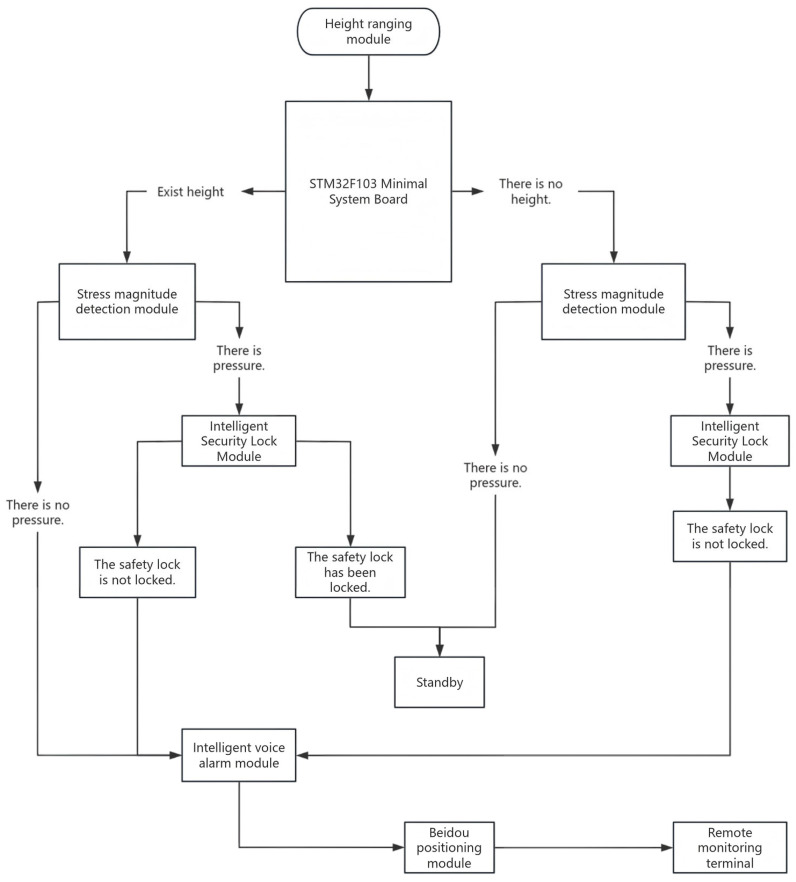
Application flowchart.

The application flowchart of the safety work suit elaborately depicts each link of safety work compliance, including initiation, response, and alarm, providing clear step-by-step instructions for on-site workers. Workers can systematically understand the functional methods of the safety work suit to ensure their own safety during the operation process. The application flowchart is shown in Figure 1.

**Figure 2:**
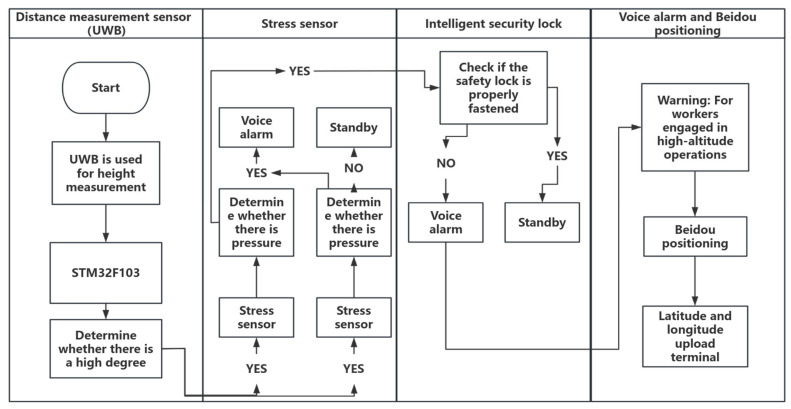
Schematic diagram of the working principle of high-altitude safety lock.

The height-ranging module installed on the safety belt is used to detect whether the workers are at a height of 2 m or more, and the stress detection module installed on the waist of the safety belt is used to monitor whether the workers are wearing the safety belt properly. When the height communication ranging module detects that the operator is at a height of 2 m or more, if the stress value detected by the stress detection device at this time is less than the set stress threshold and the time exceeds the set time threshold, the on-site intelligent voice alarm device will sound an alarm. At the same time, the intelligent safety lock module can detect whether the safety buckle of the high-altitude operator is fastened. Remind high-altitude workers to wear safety suits properly and remind the work supervisor to strengthen the control of the work site. At this time, the Beidou positioning module collects the geographical coordinates (longitude and latitude) of the system to facilitate remote monitoring personnel in controlling measures to prevent high-altitude falls on the site and accurately locate the workers, as shown in Figure 2 below.

### 2.1. System Composition and Application Principles

**Figure 3:**
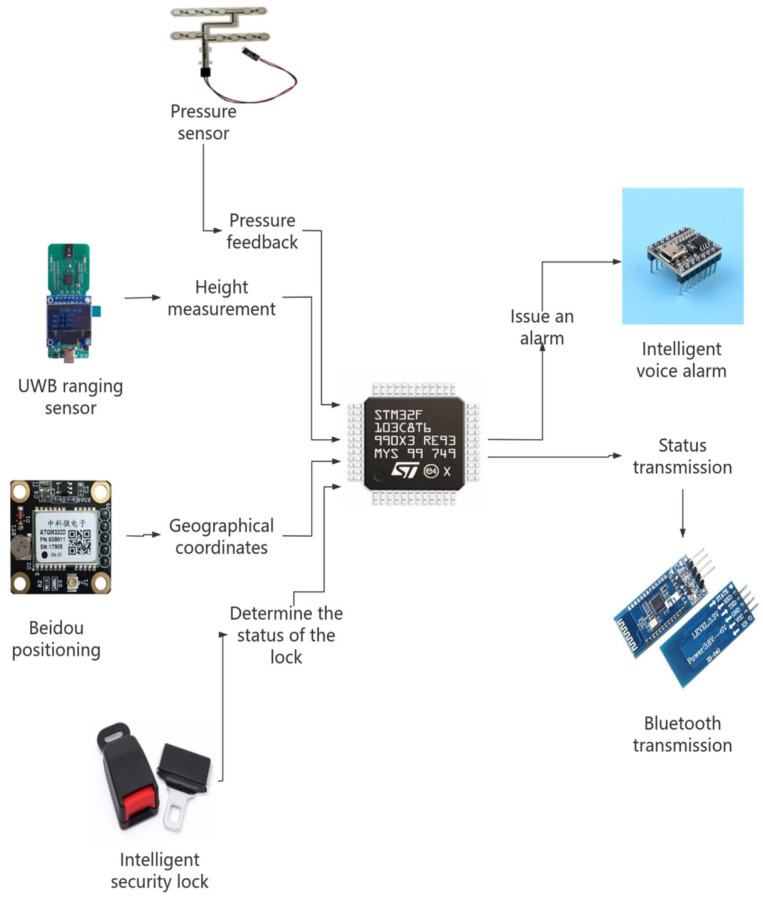
Bidirectional ranging flowchart.

This system is composed of multiple key sensors, mainly including the ultra-wideband (UWB) ranging module, stress detection module, intelligent safety lock module, intelligent voice alarm module, and other modules such as Beidou positioning. The Ultra-Wideband (UWB) ranging module employs the BP-TWR-50 model, supporting a data rate of up to 6.8 Mbps. This module is characterized by high precision and low power consumption, as well as advanced capabilities in high-precision time measurement and ranging. Additionally, it supports multipath suppression, which enhances its performance in distance measurement applications. The stress monitoring module utilizes the MD30-60 model piezoresistive thin-film sensor, known for its high sensitivity, rapid response, and robust environmental adaptability. The intelligent safety lock is independently developed, featuring a simplified mechanical structure with latch monitoring functionality. Its mechanical performance complies with international safety standards. The intelligent voice alarm system incorporates the JQ8900-16P voice chip, offering high-quality and high-volume audio output. Different voice broadcasts are controlled via single-chip microcomputer signals. The Beidou positioning system adopts the SKG12D-09H dual-frequency single Beidou module, which boasts high integration, simple peripheral application circuits, and support for raw observation measurement output. Furthermore, it includes interference detection and alarm functionalities. This article will elaborate in detail on the functional principle of sensors and their application in monitoring the status of equipment used by high-altitude workers. The application effect diagrams of each sensor are shown in Figure 3.

#### 2.1.1. UWB Module

UWB technology represents a great breakthrough in the field of positioning technology. UWB sensors utilize ultra-wideband technology for high-precision distance measurement, featuring excellent positioning accuracy and the ability to operate effectively in harsh environments with numerous obstacles, even under non-line-of-sight (NLOS) conditions [12].

When a transmission system has an instantaneous spectrum occupation close to or greater than 500 MHz or a fractional bandwidth greater than 20%, it is called UWB technology, and the relationship is expressed as follows:

(1) $$\frac{F_{H}-F_{L}}{F_{C}}\geq 20\%$$

(2) $$F_{H}-F_{L}\geq 500\mathrm{MHz}$$

$F_{H}$ represents the high cut-off frequency when the signal frequency drops to −10 dB.

$F_{L}$ is the low cut-off frequency under the same advanced conditions.

$F_{C}$ is the center frequency of the main signal energy and information and generally has the maximum power spectral density.

$$F_{C}=\frac{F_{H}+F_{L}}{2}.$$

**Figure 4:**
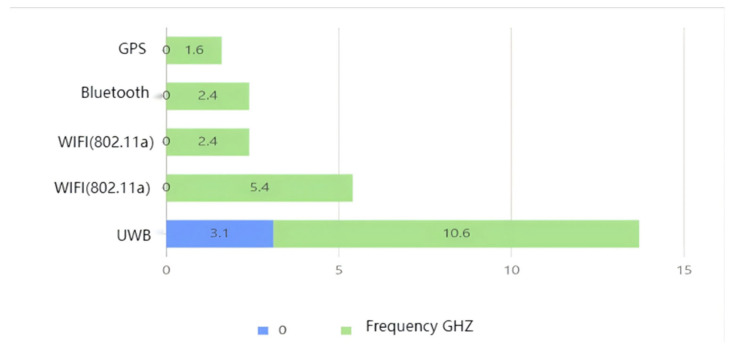
A frequency comparison chart between UWB and traditional passage technologies.

UWB technology has the characteristic of a large bandwidth and may interfere with each other and with other narrowband systems. To solve this problem, the Federal Communications Commission stipulates that a total bandwidth of 7.5 GHz between 3.1 GHz and 10.6 GHz is the UWB communication spectrum. And the Effective Isotropic Radiated Power (EIRP) is no higher than −43.1 dBm/MHz to ensure no interference to other systems. As shown in Figure 4 below, it is a frequency comparison chart between UWB and traditional passage technologies.

Among the numerous radio transmission signals, compared with common systems, the most prominent feature of UWB is its large bandwidth and low power consumption, which improves the channel capacity and transmission rate. It can be known according to the Shannon-Hartley theorem. The amount of information transmitted per unit time is proportional to the signal bandwidth, and there is an inverse relationship between bandwidth and power consumption. The relationship can be expressed as follows:

(3) $$C=BI{\mathrm{og}}_{2}(1+\frac{\bar{R}}{N})$$

C represents the information capacity in units of bit/s.

B represents the information bandwidth in units of Hz.

$\bar{R}$ represents the average received signal power on B in units of dBm.

N represents the average noise on B in units of dBm.

From the relationship, it can be known that under a specific capacity, the bandwidth can be increased to make the power consumption less. And it is easier to increase the bandwidth than to increase the Signal Noise Ratio (SNR). $\frac{\bar{R}}{N}$ is often used to represent the signal-to-noise ratio. Therefore, the power consumption of large broadband is lower, which is more conducive to the transmission of information.

Ultra-wideband (UWB) technology has shown significant advantages in the fields of high-speed communication and precise positioning due to its unique signal characteristics. UWB signals have an extremely wide bandwidth, can provide a large number of communication channels, and at the same time, the signal duration is extremely short, the time resolution is extremely high, and the wavelength is short, making it perform well in anti-attenuation [13]. Furthermore, UWB signals occupy a lower carrier frequency, making it easier for them to penetrate obstacles [14].

In the field of safety, the UWB ranging module used in this paper can precisely determine whether the workers are at a dangerous height of more than 2 m. Since in the high-altitude operations in the power grid, the majority of the maintenance heights of transmission and distribution line towers do not exceed 50 m, the power of the UWB ranging module used in this paper is relatively small. Its vertical ranging upper limit is set at 50 m, which meets the requirements of engineering applications. This is of crucial importance for the safety of high-altitude operations. UWB technology measures distance through the Time Difference Ranging (TWR) algorithm and can achieve centimeter-level accuracy, thereby realizing the safety monitoring of high-altitude workers [15].

**Figure 5:**
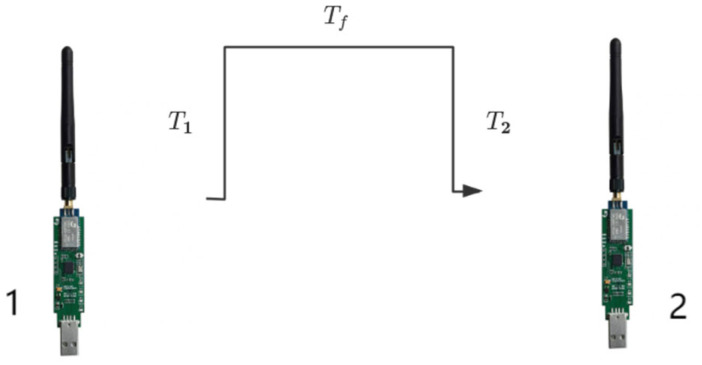
The hardware delay error of UWB.

However, the traditional TWR algorithm has certain limitations. The errors of UWB mainly fall into three aspects, namely hardware delay error, clock drift error, non-line-of-sight error, and multipath error. Among them, the hardware delay error and clock drift error are the main sources of error under the line-of-sight range. The influence of the working environment and production technology may lead to measurement errors. The hardware delay is antenna delay. The existence of the antenna causes a time difference between the signal propagation and the timing of the chip, resulting in ranging errors when calculating the distance, as shown in Figure 5.

The main body of the UWB ranging module. The main board contains a microprocessor that processes the real-time ranging signals for transmission, converting the electrical signals into real-time distance values. The black part is the ranging signal transmission antenna, which is used for the long-distance transmission and reception of signals. The hardware delay error diagram of UWB is shown in Figure 5. Distance measurement is conducted on UWB sensor 1 and UWB sensor 2. The time from when the chip of sensor 1 starts timing to when the antenna emits a signal is T1, and the time from when the antenna of sensor 2 receives it to when the chip stops recording is T2. Tf represents the true recording time of the signal. The error generated by the hardware antenna of the UWB sensor 1 is as follows:

(4) $$D_{h1}=C\times t_{1}$$

The error generated by the hardware antenna of the UWB sensor 2 is as follows:

(5) $$D_{h2}=C\times t_{2}$$

In the formula, C represents the speed of light, and $D_{h1}$ and $D_{h2}$ are the hardware delay errors of sensors 1 and 2, respectively.

Clock drift error refers to the error caused by the inconsistency between the set frequency of the sensor and the actual frequency. The ranging error *D_c_* caused by clock drift is as follows:

(6) $$D_{c}=C\times t\times (\gamma_{1}+\gamma_{2})=D\times (\gamma_{1}+\gamma_{2})$$

In the formula, $\gamma_{1}$ and $\gamma_{2}$ are the clock drift parameters of sensors 1 and 2, respectively, and D is the distance between the sensors.

It can be known from the above that the ranging error model between the two UWB sensors is as follows:

(7) $$\Delta D=\gamma \times D+D_{h}$$

In the formula, ΔD represents the ranging error, $D_{h}$ represents the hardware delay error, and γ represents the clock drift parameter.

The parameters of the error model will change with the aging of electronic components and environmental factors such as temperature, humidity, and air pressure. Therefore, in daily life, it is also necessary to update the error model parameters in real time to ensure accurate error suppression.

While achieving error suppression, we use UWB sensors without antennas. Through hardware optimization and the use of UWB sensor modules with ultra-small antennas, the hardware delay error of the sensor is greatly reduced. The distance between the ranging module (tag and base station) is calculated by measuring the propagation time of the signal. However, the clock signals of the two ranging modules do not come from the same clock source. Different clock frequencies can lead to deviations in the recorded moments and clock drift errors, thereby resulting in inaccurate calculated distances [16]. To solve this problem, the SDS-TWR method was proposed to reduce the requirement for high-precision clock synchronization [17].

**Figure 6:**
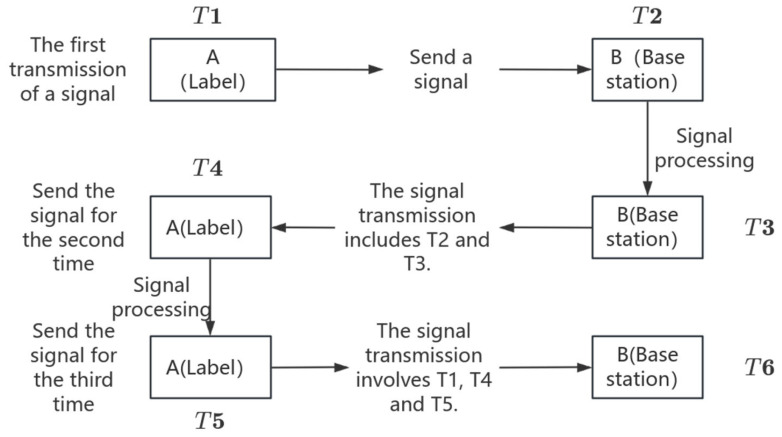
Bidirectional ranging flow chart.

The UWB ranging algorithm of this device uses Double-Sided Two-Way Ranging for bidirectional ranging, which is an improved protocol of TWR [18]. Through three message exchanges and symmetrical time measurements, there is no need for hardware clock synchronization, significantly reducing the error caused by inconsistent clock frequencies. The range is more accurate. The bidirectional ranging flowchart is shown in Figure 6.

In this context, T denotes the present time at which the signal is transmitted. The subsequent computation is performed to determine the transmission duration.

The round-trip time of signal A is as follows:

(8) $$T_{7}=T_{4}-T_{1}$$

The signal-processing time of A is as follows:

(9) $$T_{8}=T_{5}-T_{4}$$

The round-trip time of signal B is as follows:

(10) $$T_{9}=T_{6}-T_{3}$$

The signal-processing time of B is as follows:

(11) $$T_{10}=T_{3}-T_{2}$$

The actual transmission time of the signal is as follows:

(12) $$TOF=\frac{T_{7}\times T_{9}-T_{10}\times T_{8}}{T_{7}+T_{9}+T_{10}+T_{8}}$$

TOF stands for Time of Flight.

Therefore, the true vertical distance D between the ground and the personnel working at high altitudes, measured by UWB, is given by $D=\frac{TOF\times C}{2}$. (The unit of D is meters per second, m/s, $c=3\times 10^{8}m/s$.)

UWB ranging may be subject to various environmental interferences. For instance, during high-altitude operations, the surrounding environment can be complex, which may cause interference to UWB ranging, including multipath interference, material attenuation, and non-direct diffraction. Multipath interference refers to the UWB signal being reflected and scattered by objects during its journey from the transmitting end to the receiving end, propagating through multiple paths. These signals superimpose with the direct signal, potentially misidentifying strong reflected signals as direct signals, resulting in an overestimation of the ranging distance, or distorting the shape and arrival time of the direct pulse, increasing the difficulty in determining the starting point of the detection pulse. This can be addressed by applying the first arrival path detection algorithm to estimate the earliest arrival signal time and using the super-resolution algorithm to estimate the multipath components to improve accuracy. Material attenuation works on the principle that when UWB signals penetrate different materials, their energy is absorbed, reflected, and scattered, causing a significant attenuation in signal strength, making the signals weaker, increasing the bit error rate, and even making the signals undetectable. This can be resolved by designing the link budget and reserving the transmission power margin and receiver sensitivity for most of the impacts. Non-direct diffraction may lead to positive deviations in the ranging results, with weak and unstable signal strength. This can be determined by analyzing the characteristics of the received signal to identify the NLOS state, reducing or eliminating the NLOS ranging value, and further improving the measurement accuracy. The ultra-wideband characteristic of UWB has significant advantages in terms of accuracy and anti-interference. Although it may have some impact in complex environments, its measurement values still fall within the error range for high-altitude safety ranging.

#### 2.1.2. Stress-Sensing Module

In fall accidents, the landing posture of the human body is mostly supine or prone, among which the impact force borne by the abdomen (waist) is relatively large, which provides a key basis for the design of the stress-sensing module [19]. Thin-film pressure sensors for pressure monitoring are mainly divided into alloy thin-film pressure sensors and semiconductor thin-film piezoresistive sensors. In the safety belt stress detection module, the thin-film piezoresistive sensor is widely used due to its high sensitivity and repeatability [20,21].

Silicon and polysilicon are commonly used piezoresistive materials in piezoresistive pressure sensors for micro-electromechanical systems (MEMS). However, the application of silicon-based sensors is limited in harsh environments such as corrosive media, high radiation, and high temperatures. Silicon carbide (SiC) has become an ideal choice for harsh environments due to its excellent thermal and mechanical properties. In scenarios where it is necessary to monitor the force on the safety belts of workers, MEMS pressure sensors based on silicon carbide can reliably detect stress values, determine whether workers are wearing safety belts, and whether the safety belts are subjected to sufficient tension, thereby ensuring the safety status of workers. In addition, SiC materials are also highly suitable for high-altitude applications.

The high-altitude safety device uses a thin-film piezoresistive sensor, and its main components include the substrate, the conversion element, and the signal conditioning circuit [22,23]. The principle is that the thin-film pressure sensor can measure the magnitude of a specific force. Through the electrical signal changes caused by the pressure, after calibration, it is converted into a force value, and the force is measured through the “pressure-area” conversion. However, it must meet the conditions such as “clear force area, accurate calibration, and controllable error”. In practical applications, through reasonable structural design and signal processing, its force measurement accuracy can reach ±1% to ±5%, and it is suitable for various scenarios ranging from mN to kN. This type of sensor usually contains a temperature sensor for temperature compensation, as temperature may affect the accuracy of the sensor.

**Figure 7:**
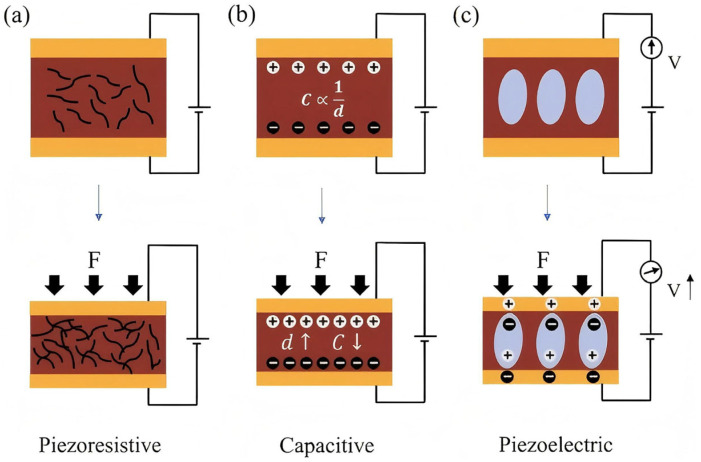
Schematic diagram of pressure sensor transduction mode (**a**) piezoresistive type; (**b**) Capacitive type; (**c**) Piezoelectric type.

Figure 7 is a schematic diagram of the transduction mode of pressure sensors, presenting three common pressure sensing mechanisms: piezoresistive, capacitive, and piezoelectric. Figure 7a shows piezoresistive, Figure 7b shows capacitive, and Figure 7c shows piezoelectric. The device adopts the piezoresistive sensing method shown in Figure 7a, illustrating how the conductive path deforms when a force is applied, thereby changing the resistance of the material [24].

**Figure 8:**
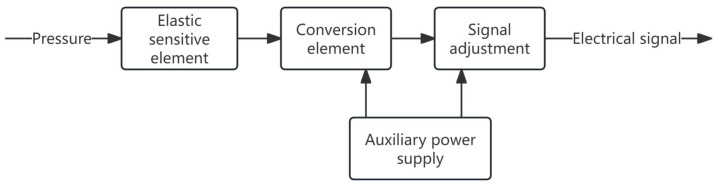
Schematic diagram of the functional modules of the thin-film pressure sensor.

The main components of thin-film piezoresistive sensors include the base, conversion elements, signal conditioning circuits, etc. The thin-film resistance layer generates corresponding resistance changes by sensing the strain of the elastic element, and outputs the corresponding voltage signal through the signal conditioning circuit, thereby completing the conversion from non-electrical quantity to electrical quantity. The functional module schematic diagram of the thin-film pressure sensor is shown in Figure 8.

The thin-film piezoresistive sensor, whose piezoresistive effect stems from the change in the band structure under the action of stress, leads to changes in the carrier mobility and concentration, and subsequently causes a change in resistance. Its piezoresistive behavior follows the following basic relationship formula:

(13) $$\frac{\Delta R}{R}=\Pi_{ijkl}\times \sigma_{ki}$$

where ΔR/R is the relative change in resistance, $\Pi_{ijkl}$ is the piezoresistive coefficient tensor (Pa^−1^), and $\sigma_{\mathrm{ki}}$ is the stress component (Pa). For the cubic crystal system, the piezoresistive coefficient is mainly described by the longitudinal piezoresistive coefficient and the transverse piezoresistive coefficient, and its values vary significantly with the doping concentration and temperature. The crystal orientation and stress state of the film directly affect the effective component of the piezoresistive coefficient. The physical principle of the piezoresistive SiC stress sensor based on the film lies in the coupling of the piezoresistive effect and mechanical behavior. Under dynamic loads, the propagation and resonance characteristics of strain waves need to be considered, and the thermal effect affects the measurement accuracy through the temperature dependence of the piezoresistive coefficient and thermal stress. Through material design and structural optimization, the performance of the sensor in extreme environments can be significantly improved.

The calculation formula of the thin-film pressure sensor is:

(14) $$U_{0}=(1+R_{AO-RES}\times \frac{1}{R_{X}})\times 0.1$$

In the formula, $U_{0}$ represents the voltage value output by the sensor module, $R_{AO-RES}$ is the magnitude of the feedback resistance, and $R_{X}$ is the output resistance of the thin-film pressure sensor.

The feedback resistance of the sensor is 10 kΩ. From the pressure resistance curve graph of the sensor, the relationship between $\frac{1}{R_{X}}$ and F (pressure/kg) is as follows:

(15) $$\frac{1}{R_{X}}=0.01\times F+0.0016$$

**Figure 9:**
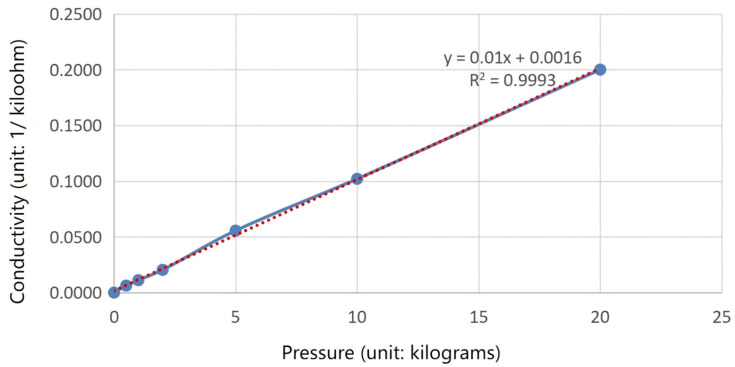
The relationship between the applied pressure and the electrical conductivity.

The relationship between the applied external pressure and the conductance is shown in Figure 9, the blue curve in the figure represents the corresponding conductivity data points measured under different pressures, and the red curve represents the fitting curve of the corresponding conductivity data points measured under actual conditions.

Substituting $R_{AO-RES}$ and $\frac{1}{R_{X}}$ into the formula for the output voltage of the sensor gives us the following:

(16) $$U_{0}=\left[1+10\times \left(0.01\times F+0.0016\right)\right]\times 0.1$$

The actual pressure value F can be converted from $U_{0}$ collected by the single-chip microcomputer, and the single-chip microcomputer judges its pressure feedback.

This device arranges the stress sensors at the L3–L5 vertebrae positions at the posterior lumbar region, which is the optimal choice based on the unique biomechanical characteristics of this area. According to the research data from the Nahum impact dynamics model, this position will bear approximately 78% of the total impact load during a fall impact. At the same time, the muscle tissues in this area can provide good impact buffering, and these characteristics make this position the best choice for stress monitoring [25].

In terms of sensor adaptability, the posterior lumbar region shows significant advantages. The body surface temperature fluctuation at this location is extremely small, with a change of no more than 2 °C per hour. This characteristic enables sensors made of SiC material to maintain a measurement accuracy of ±0.5% FS, jointly ensuring the accuracy and reliability of the measurement data.

The force on the waist is significantly correlated with the body’s backward inclination angle, and this relationship can be quantitatively analyzed through a formula:

(17) T=(G×h)÷(2d)×tanθ

*T* represents the force on the waist (N), *G* represents the gravity corresponding to the body weight (N), *h* represents the height of the center of gravity (m), *d* represents the horizontal distance from the waist support point to the foot support point (m), and is the backward inclination angle of the body (°). Taking an adult weighing 70 kg as an example, the calculation shows that when the backward inclination angle is 15°, the force on the waist is approximately 160 N, which is within the safe range; when the angle increases to 30°, the force rises to 340 N, which is close to the safety upper limit. Therefore, the range of the pressure sensor in this paper is selected as 500 g–50 kg. When the high-altitude workers are in a suspended state, in the normal working state, the pressure sensor is about 160 N, which is higher than the set threshold value of 20 N, and can operate normally and transmit the pressure signal to the main control chip.

#### 2.1.3. Other Modules

The Beidou positioning module provides real-time geographical locations of the operators, ensuring that their positions can be accurately located in emergency situations.

The intelligent safety lock module detects whether the safety lock is correctly installed through the lock clip sensor [26]. If it is found that the safety lock is not worn correctly, the system will immediately issue an alarm to remind the operator to make adjustments.

The voice alarm module uses the JQ8900-16P voice chip. When the system detects potential safety risks, it will issue a voice alarm to ensure that the operators are warned in the first place and can take timely measures.

#### 2.1.4. Stability and Adaptability Analysis of the System in Different Environments

In outdoor and high-altitude environments, thermal changes and electromagnetic interference have significant potential impacts on the performance of the sensors. In terms of thermal changes, due to the temperature-dependent drift of material parameters, such as the resistance value of metals and the threshold voltage of semiconductor devices, along with the sharp temperature differences and low heat dissipation efficiency in high-altitude areas, and the problem of thermal gradients and thermal fatigue caused by multi-module integration, electromagnetic interference stems from frequent lightning activities and high-voltage power lines in high-altitude areas, which affect the sensors through radiation and conduction coupling, and the digital circuits in multi-module integration will interfere with the analog circuits.

Therefore, this paper adopts heat drift suppression strategies such as low-temperature coefficient materials, active cooling, and temperature compensation circuits, as well as electromagnetic interference shielding methods such as circuit filtering and grounding optimization. At the same time, it conducts collaborative design for heat dissipation and air pressure in high-altitude environments and material anti-aging treatment, and also needs to pay attention to the cross-influence of thermal and electromagnetic interference, and design modular partitioning to ensure the stability and reliability of the sensors in extreme environments.

### 2.2. Innovation Points of Module Application

The intelligent high-altitude operation device of this project has realized real-time height monitoring by UWB technology. It has also designed new safety latches and other innovative points, significantly enhancing the safety of operations, effectively reducing the incidence of accidents, and improving the monitoring efficiency. These advantages have promoted technological innovation in the field of high-altitude operations and enhanced the level of safety guarantee.

#### 2.2.1. UWB Real-Time Height Monitoring

This project utilizes ultra-wideband (UWB) ranging technology and adopts the Double-Sided Two-Way Ranging (DSTWR) method. By employing this method, the precise measurement of the vertical distance between two points has been achieved, significantly reducing the ranging error. High-altitude workers can observe the exact height from the ground in real time, enabling them to realize that they are at a dangerous height without proper protective measures, thereby enhancing their safety awareness.

#### 2.2.2. New Type Safety Snap-On Connector

This project proposes an innovative and convenient mechanism for putting on and taking off traditional work clothes—a new type of snap fastener system. The rear end of the snap tongue is connected to the woven belt of the safety belt, and ensures that the interface complies with international standards. The snap fastener is integrated with a stable locking mechanism inside, where the lock hook is a small metal hook that tightly bites into the snap tongue. At the same time, a spring system is introduced as a dual locking guarantee. This snap fastener enables quick fastening and unfastening in the air, and also ensures the safety of the snap fastener, which will not be easily released. In case of danger for the workers in the air or in extreme situations, they can quickly remove the safety belt and fasten it again. Compared with traditional safety belts, it achieves a win-win situation of convenience and safety.

#### 2.2.3. Precise Collaborative Positioning

High-precision seamless positioning technology is adopted, and the Beidou satellite positioning system is combined with Bluetooth technology [27]. Achieve precise division of the construction operation area. Managers can flexibly demarcate dangerous areas for high-altitude operations on the management platform based on Beidou positioning coordinates and Bluetooth beacons. This system adopts a triple positioning guarantee mechanism. Firstly, Beidou satellite positioning provides a wide-area position reference. Secondly, Bluetooth low-latency communication achieves precise positioning of regional boundaries. Finally, Bluetooth beacons assist in seamless positioning both indoors and outdoors. This multi-technology integrated positioning solution ensures positioning accuracy and real-time early warning, providing reliable safety guarantees for high-altitude operations.

#### 2.2.4. Intelligent Voice Warning System

The intelligent voice warning system based on the JQ8900-16P voice chip realizes multi-level voice warning functions through preset voice library and real-time synthesis technology. When the intelligent safety belt equipped with the Beidou positioning module detects that the workers enter the dangerous area, the system immediately triggers a voice alarm, clearly indicating “Please fasten your safety belt”, effectively enhancing the safety vigilance of the workers. This voice warning system is deeply integrated with the Beidou high-precision positioning technology, significantly improving the real-time accuracy of construction safety management. It not only enhances the safety awareness of the workers but also effectively prevents violations such as not wearing safety belts correctly, providing more reliable safety guarantees for high-altitude operations.

#### 2.2.5. Small in Size, Low in Power Consumption, and Stable in Performance

**Figure 10:**
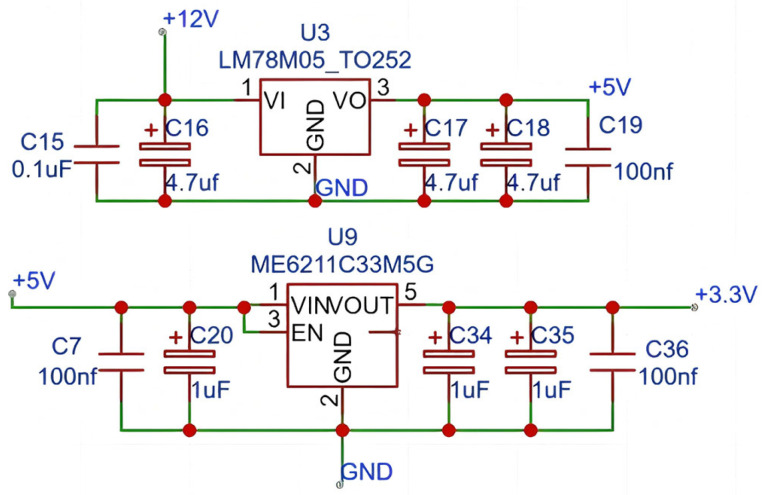
Schematic Diagram of Power Supply Module.

This project adopts the STM32F103C8T6 microcontroller as the main control chip. This chip has a high main frequency of 72 MHz, with high computing efficiency and low power consumption, which is suitable for real-time control tasks. It also has abundant peripheral resources. It also supports mainstream development tools such as STM32CubeMX 6.14.0 (graphical configuration tool) and Keil uVision5, and also supports low-power mode, suitable for battery-powered applications. At the same time, a set of low-cost, low-power, and small-volume power supplies is used. The power supply adopts two linear voltage regulators to achieve 5 V and 3.3 V power supplies, which can save peripheral devices and realize the advantages of a small volume. It is suitable for applications with batteries. The schematic diagram of the power supply module is shown in Figure 10.

The LM78 series of linear regulators offers a variety of fixed output voltages, making them suitable for numerous application scenarios. They also feature current limiting, thermal shutdown, and safety zone protection functions, making them robust and durable. The ME6211 series of regulators boasts low cost and high stability. This high level of stability ensures voltage stability even during frequent load fluctuations. The stable power supply supports a wide voltage input and can still be used when an appropriate power source cannot be found. It has low power consumption while also extending the service life of the device and protecting it. The stable power supply ensures the long-term stable operation of the device and continuous protection for high-altitude workers.

## 3. Simulation Operation and Experimental Verification of High-Altitude Operation Status

To verify the actual operational effectiveness of the intelligent high-altitude work safety system, this paper conducted Proteus 8.17 Professional simulation and test experiments. Proteus is a powerful EDA tool developed by Lab Center Electronics Company in the UK. It integrates the functions of circuit simulation software, PCB design function, and virtual model simulation software, and holds an extremely important position in the field of electronic design. Using ProSPICE function mixed simulation in Proteus can achieve the mixed simulation of digital and analog circuits. In this design, the Protues software was used to build an analog high-altitude work platform, simulate different working scenarios, and conduct comprehensive tests on the various functions of the system through test experiments.

### 3.1. State Determination Logic Simulation

**Figure 11:**
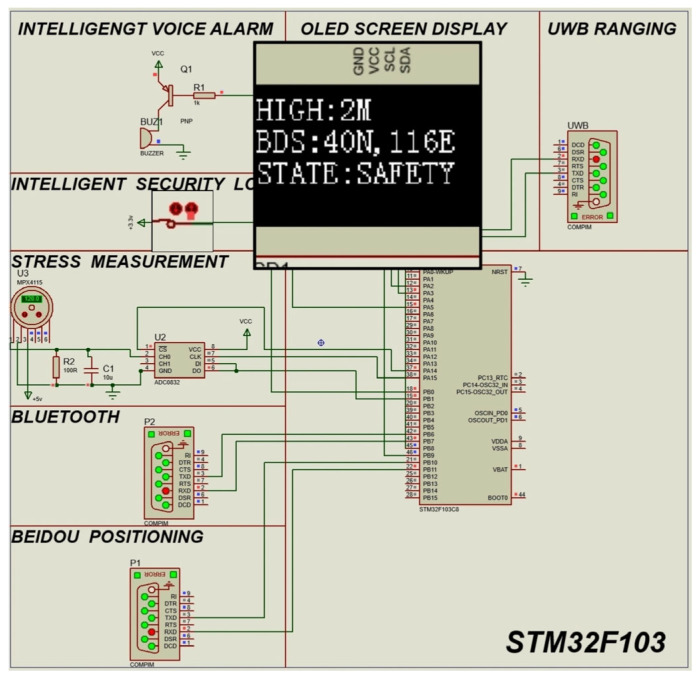
Simulation diagram of intelligent safety locking during high-altitude operations.

**Figure 12:**
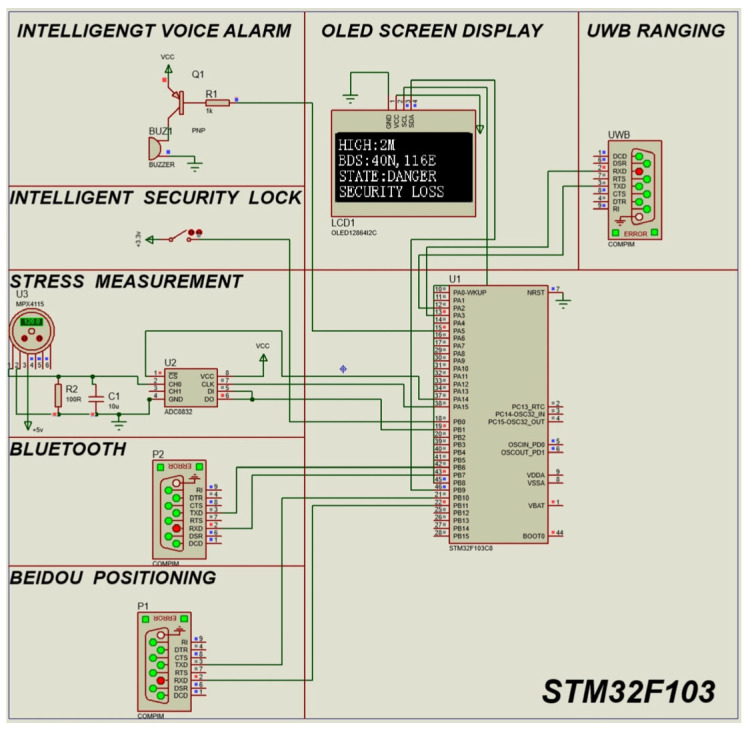
Simulation diagram of intelligent safety lock disconnection during high-altitude operations.

First, view the serial port data transmitted back by UWB, Bluetooth, and Beidou positioning through the upper computer. In Protues, the serial port data is transmitted to the single-chip microcomputer through the virtual serial port. Drive the buzzer with a transistor to give R1 a low-level buzzer, and imitate the intelligent voice alarm system through the buzzer. The pressure sensor MPX4115 (NXP Semiconductors N.V., Eindhoven, The Netherlands) is used to imitate the stress sensor, and then the pressure signal is measured through ADC0832 (Texas Instruments Incorporated, Dallas, TX, USA). Simulate the intelligent security lock through a physical switch to achieve opening and closing. In this way, through different combinations of modules, a simulation circuit is formed. The simulation diagrams are shown in Figure 11 and Figure 12.

Figure 11 shows the real-time simulation interface of the intelligent safety lock during high-altitude work: the lock body is tightly engaged with the safety rope, and the green status indicator light keeps flashing. The upper half of the OLED display presents the geographic coordinates [2M] [40° N 116° E] in double-line characters, and the lower half is dynamically refreshing with [SAFETY]. The positioning accuracy reaches ±0.1 m. The data from the environmental monitoring module remains stable within the safety threshold (wind speed < 5 m/s, inclination angle < 8°), and no alarm sounds have been triggered. It complies with the requirements of GB/T 3608-2008 High-altitude Work Safety Standards [28].

Figure 12 Dynamic display of the simulation system: When the smart safety lock worn by the operator is in an open state, the red status indicator light keeps flashing. The Beidou positioning module carried by the operator continuously uploads real-time coordinates (40° N, 116° E). At this time, the high-altitude work platform has been raised to the critical height of 2 m, and the safety monitoring center immediately activates the third-level warning mechanism—the device buzzer emits intermittent alarms of 96 decibels, the OLED display presents a red flashing border, and the center displays the bilingual warning message “Danger! DANGER! The locking device is not effective” in the middle, warning the operator and providing real-time reminders to the auxiliary operators on the ground who are conducting supervision.

The simulation uses mechanical switches to simulate the opening and closing of the intelligent security lock. Through serial communication (Serial Communication), the UWB ranging module, the Beidou module, and the returned binary data are processed. Then, Bluetooth is used to output the current status to the mobile phone for display, so that the ground personnel can monitor it. At the same time, OLED can display the current height of the high-altitude worker relative to the ground, and the current latitude and longitude of the worker, so that the high-altitude worker and the background can confirm the current location. STATE represents the current status of the current worker. By displaying DANGER and SAFETY, it reminds the high-altitude worker to pay attention to dangerous operations.

**Figure 13:**
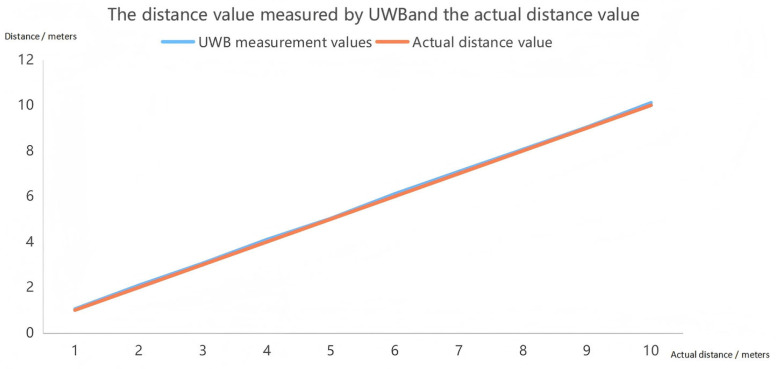
Comparison Chart of Actual Measurement Values and UWB Measurement Values.

The simulation also verified the ranging algorithm of UWB (DS-TWR), and displayed the returned height on the screen. The measured values of UWB ranging were compared with the actual values as shown in Figure 13.

**Figure 14:**
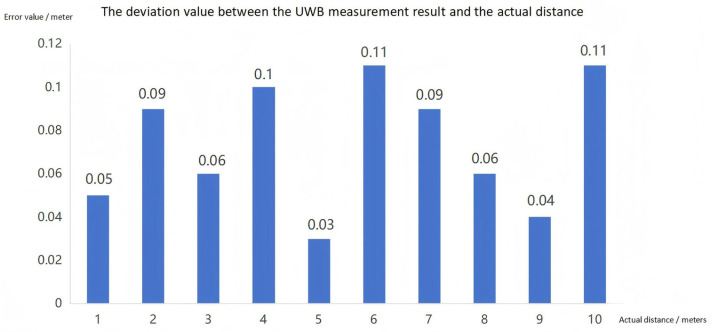
Error Values of Actual Measurement and UWB Measurement.

The UWB ranging measurement and the actual measurement error values are shown in Figure 14.

By comparing the actual measured distance with the data obtained through UWB ranging technology, the study found that the error of UWB ranging technology is relatively small, and its ranging accuracy meets the application requirements. Compared with Bluetooth and Wireless Local Area Network (WIFI) technologies, UWB ranging technology has achieved a qualitative leap in accuracy.

Ultra-wideband (UWB) technology has received extensive attention in indoor positioning systems due to its outstanding ranging accuracy [29]. Compared with other wireless technologies such as Bluetooth and WIFI, UWB transmits data using ultra-short pulses over a wide spectrum, giving it a significant advantage in time resolution and enabling positioning accuracy at the centimeter level [30].

The advantage of UWB ranging technology lies in its high precision. The outstanding time resolution of UWB enables it to achieve precise distance measurement. Studies have shown that the ranging accuracy of commercial UWB modules can be less than 10 cm [31]. Compared with traditional positioning systems based on WIFI, Bluetooth, and GPS signals, UWB has several times higher accuracy [32].

**Figure 15:**
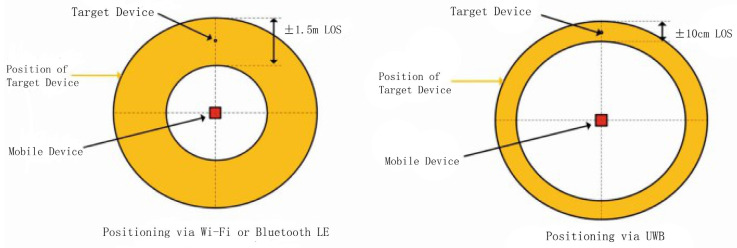
Comparison Chart of Positioning Accuracy for WiFi/BLE and UWB.

Figure 15 compares the positioning accuracy of WiFi/Bluetooth and UWB, clearly demonstrating the superiority of UWB in terms of positioning accuracy [33].

### 3.2. Experimental Verification

We conducted mechanical performance tests, sensor function tests, and environmental adaptability tests on this device. During the mechanical performance test, we used a tensile machine to apply gradually increasing static tension to key parts such as the safety belt, connection ring, and intelligent safety lock, applying at least 15 kN of force, to test their static ultimate load. Simultaneously, dynamic impact tests were also carried out. After the tests, all the components of this device met the standards specified in Table 1.

In the sensor function tests in Table 2, we adopted the control variable method to conduct the sensor function tests. During the tests, we first divided the tests into two different states: one is the state with a height greater than two meters, and the other is the state with a height less than two meters. We conducted function tests on each sensor under different states to determine whether each sensor could operate normally.

Firstly, when the height is less than 2 m, it operates according to normal logic. When the height is less than 2 m and the stress sensor has no pressure, it will not issue an alarm. When the stress sensor detects pressure and the measured stress is greater than the 20 N threshold, while the safety lock is not engaged, a danger alarm will be triggered. However, when the force is less than 5 N, it can be regarded as a situation where the waist has no stress monitoring. At this time, the mechanical effect is small and the device is in a relatively relaxed state, so it will not malfunction. We conducted 500 consecutive tests. Through the control of a single variable of the intelligent safety lock and the stress monitoring sensor, each sensor was tested separately. The results are shown in Table 2 below.

When the height exceeds 2 m, it operates according to normal logic. When the height exceeds 2 m and the stress sensor has no pressure, an alarm will be triggered. If the stress sensor has pressure and the safety lock is not fastened, a danger alarm will be triggered. We conducted 500 consecutive tests and tested each sensor separately through the control of a single variable. The results are shown in Table 3 below.

Based on the above experimental data analysis, each module can operate independently. Through actual data analysis, the monitoring probabilities are all above 99%. In practical applications, it can effectively ensure the safety of workers performing high-altitude operations and provide a solid safety barrier.

## 4. Research on System Integration Optimization and Adaptability to Practical Applications

**Figure 16:**
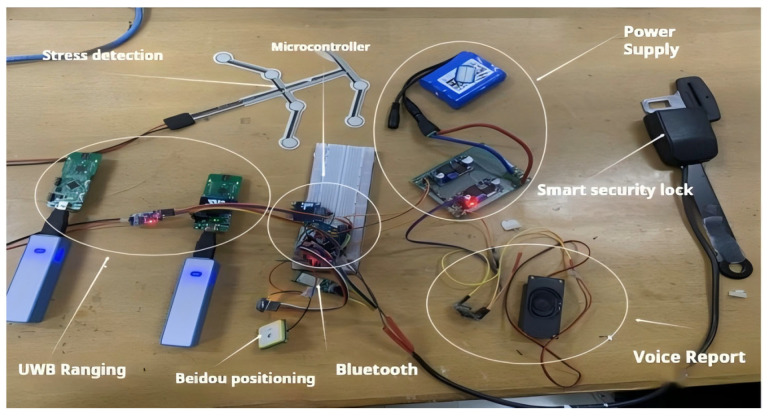
Unintegrated Circuit Diagram.

The integration diagram of the device’s components is shown in Figure 16. In the initial stage of production, the components were made in separate modules and then combined. At that time, they were connected using DuPont wires. The current version has achieved the operation of the core functions, but it is larger in size after production. In terms of user experience and system stability, further optimization is still needed.

**Figure 17:**
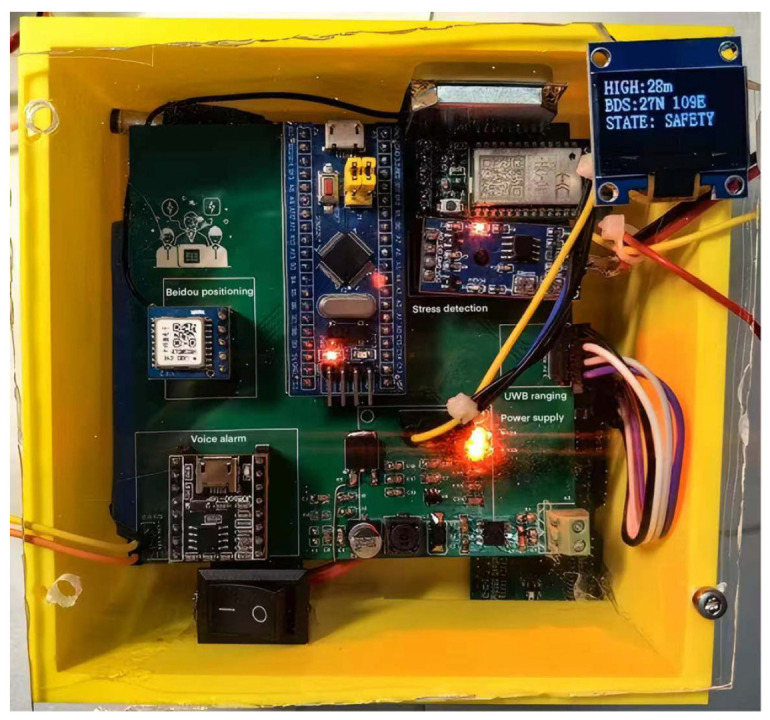
Integrated Model Diagram after Module Optimization.

As shown in Figure 17, the integrated model after module optimization is the latest version. It has undergone sensor integration optimization processing. The final board size is 10 cm × 10 cm. Integrating all sensors is achieved on one board, which realizes the advantages of small size and stable connection. A yellow shell with a length of 11 cm, a width of 11 cm, and a height of 5 cm was added using 3D printing technology. The opening on the right upper corner of the cover is 5 cm × 1 cm. The display screen can be exposed to facilitate staff viewing the information. The total weight is 200 g. It has the characteristics of being lightweight, sturdy, and durable.

This system has implemented two fault detection methods. The first one is physical damage detection: observe whether there are cracks, deformations, or water ingress traces on the device shell, and whether there is obvious hardware damage, such as burnt circuits, component detachment, or capacitor bulging on the internal circuit board. Check whether the connection wiring harness is loose or oxidized, and test whether it recovers after re-plugging the interface. The second one is using indicator lights and display feedback: record the status of each indicator light; if the power indicator light is not on, it may be a fault in the power module, the fuse has blown, or the external power supply is abnormal; if the operation indicator light flashes abnormally, it may be that the main control chip has crashed or the program has crashed; if the communication indicator light is not on or frequently reports errors, it may be a fault in the communication module or the signal antenna is damaged.

The integration and structural optimization of work suits and devices, as well as the optimized design of the embedding position of sensors on work suits, take into account wearing comfort, sensing accuracy, and structural rationality. The integrated safety device not only needs to ensure the normal operation of each module, but also needs to take into account the comfort of the operators and the convenience of operation.

The sensor should be positioned as follows based on personnel comfort and operational convenience: The UWB module needs to be connected to the shoulder or chest of the operator to ensure ranging accuracy; the stress detection module is integrated at the waist of the seat belt to monitor the force condition of the seat belt. The intelligent safety lock module and the voice alarm module are set in convenient positions on the work clothes to ensure that the workers can be promptly alerted in case of emergency. In the module embedding design, the requirements of ergonomics and sensor performance have been fully considered. To ensure the accuracy of monitoring and the comfort of wearing, the UWB module and the stress detection module are, respectively, embedded in the back and waist of the work suit, while the Beidou positioning module is also placed on the back, avoiding external interference and ensuring stable signal reception. By optimizing the structure and materials of the work suits, the work suits can ensure the performance of the sensors without adding excessive weight or a sense of restraint, thereby improving the comfort and work efficiency of the workers.

## 5. Conclusions

In this study, the UWB ranging technology is innovatively applied to the high-altitude ranging system, combined with new technologies such as new safety buckles, to improve the stability of the system operation, and an intelligent high-altitude work safety device is designed, which uses multi-sensor data fusion and real-time data analysis technology to lead the transformation of high-altitude work safety protection from the traditional passive to the active early warning mode.

For the future development of the system, we will focus on the following three R & D and testing directions: first, enhance environmental adaptability, and improve the operational reliability of the system in extreme temperature and humidity environments by designing efficient heat dissipation systems, heating modules, and moisture-proof measures; Secondly, the implementation of industry-customized design, according to the specific needs of different industries such as electric power, construction, petrochemical, etc., the development of modules with anti-interference, visual recognition, explosion-proof, and other functions to enhance the practical application efficiency of the system; Finally, it is proposed to further improve the economy of the system by adopting domestic substitution components and version grading strategies.

The intelligent aerial work safety device system will develop in the direction of intelligence and collaboration, realize the prediction function of fall risk by embedding lightweight artificial intelligence algorithms, build a multi-personnel collaborative monitoring platform combined with 5G network technology, and explore innovative applications such as augmented reality (AR) remote guidance. The technology will form an intelligent safety network integrating real-time monitoring, early risk warning, and emergency response. With the continuous optimization and industry adaptation of this technology, the safety level of aerial work will be significantly improved, and solid technical support will be provided for intelligent and safe operation.

## Figures and Tables

**Table 1:** Mechanical performance test results.

Standard Type	International Standard	Chinese Standard	Test Focus	Determine Whether It Is Qualified
Static load	EN 361 [34]	GB 6095-2021 [35]	The load-bearing capacity of the webbing and metal fasteners	Yes
Dynamic impact	ANSI Z359.1 [36]	GB/T 6096-2020 [37]	Peak impact force and energy absorption	Yes

**Table 2:** Test data of each sensor with a height less than 2 m.

	Intelligent Security Lock	Stress Measurement Value	Stress Detection Sensor	Voice Alarm	Number of Incorrect Executions	Detection Probability
Located at ground level (with a height of less than 2 m)	Lock it up	>20 N	There is stress.	Do not call the alarm	2	99.6%
	Lock it up	<5 N	No stress	Alarm	1	99.8%
	Do not lock	>20 N	There is stress.	Alarm	1	99.8%
	Do not lock	<5 N	No stress	Alarm	1	99.8%

**Table 3:** Test data of each sensor with a height greater than 2 m.

	Intelligent Security Lock	Stress Measurement Value	Stress Detection Sensor	Voice Alarm	Number of Incorrect Executions	Detection Probability
Located at ground level (with a height greater than 2 m)	Lock it up	>20 N	There is stress.	Do not call the alarm	3	99.4%
	Lock it up	<5 N	No stress	Alarm	2	99.6%
	Do not lock	>20 N	There is stress.	Alarm	1	99.8%
	Do not lock	<5 N	No stress	Alarm	2	99.6%

## Data Availability

Data are contained within the article.
